# Sudden Deterioration of a Young Patient During Elective Cesarean Section. Amniotic Fluid Embolism… or Else? – A Case Report

**DOI:** 10.2478/jccm-2024-0001

**Published:** 2024-01-30

**Authors:** Ioana Roxana Codru, Marian Valeriu Codru, Bogdan Ioan Vintilă, Ioana Gherman, Dragoș Popescu

**Affiliations:** Faculty of Medicine, Lucian Blaga University of Sibiu, Romania; Anesthesia and Intensive Care Clinic, Sibiu Emergency County Hospital, Romania; Obstetrics and Gynecology Clinic, Sibiu Emergency County Hospital, Romania

**Keywords:** cardiac, respiratory, deterioration, anaphylaxis, C-section, spinal anesthesia

## Abstract

Sudden respiratory and circulatory collapse during or immediately after delivery, vaginal or surgical, can have many causes that can lead to poor maternal outcomes. A pregnancy-induced amniotic fluid embolism and anaphylaxis are two distinct medical conditions that appear similar clinically but have very different underlying mechanisms and treatment approaches. Amniotic fluid embolism is a rare but life-threatening obstetric emergency that leads to a systemic inflammatory response that can be easily confounded with an anaphylactic reaction. We report the case of a patient with no comorbidities or allergies before the current pregnancy that was proposed for delivery by C-Section under spinal anesthesia. After delivery of the placenta and administering the test dose of antibiotic, the patient developed sudden circulatory collapse, altered neurological status, and critical respiratory distress. At that point, the two presumed diagnoses were amniotic fluid embolism and anaphylaxis. Concurrently with the diagnostic pathway, supportive measures (intubation, mechanical ventilation, hemodynamic support) were taken. The clinical evolution was favorable, and after day three, the patient was discharged from the hospital. Our case highlights the significance of promptly distinguishing between anaphylaxis and amniotic fluid embolism to facilitate the timely management of the critical situation.

## Introduction

Sudden respiratory and circulatory collapse during or immediately after delivery, vaginal or surgical, is a clinical condition that can lead to poor maternal outcomes, even maternal demise. Several conditions can lead to cardiovascular and respiratory collapse, making a timely diagnosis difficult. Taking corrective measures and establishing appropriate supportive therapy is essential in these circumstances. Moreover, coordinated action and effort of gynecologists, anesthesiologists, and neonatologists to minimize maternal and fetal morbidity and mortality is paramount.

Many conditions may appear intrapartum or immediately after delivery that can progress to severe cardiorespiratory collapse: massive postpartum hemorrhage due to unusual placentation, high parity, placental abruption, or even uterine rupture; pulmonary embolism, peripartum cardiomyopathy, myocardial infarction, aortic dissection or toxicity of administrated drugs (local anesthetics, magnesium overdose, etc.) [1]. One of the most severe complications of this period is amniotic fluid embolism (AFE), as it results in maternal death in up to 80% of the cases [2,3].

We present the case of a parturient who developed circulatory and respiratory collapse during cesarean section delivery.

## Case presentation

We present the case of a 36-year-old lady who was admitted to our hospital’s Obstetrics ward to be prepared for surgical delivery. She was G2P2, 37^+1^ weeks gestation, cephalic presentation, no labor, diabetic (Pregnancy Diabetes Mellitus), with Hydramnios, Incompatibility in the Rhesus system with isoimmunization, and obese. Besides being overweight, before the current pregnancy, the patient had no comorbidities or allergies; she never received blood products and had a previous vaginal birth that went smoothly.

She was proposed for delivery by C-section under spinal anesthesia. The patient was euglycemic on the morning of the surgery, with normal blood pressure, heart rate, and rhythm. She was premedicated with IV ranitidine and ondansetron, and she received 500 ml of normal saline on a large-bore peripheral cannula (18 G). The anesthetic technique went well: unique lumbar puncture, 27 G pencil point needle with 0.5% hyper-baric bupivacaine (12.5 mg) combined with Fentanyl (10 mcg). A grade IV motor block on the Bromage Scale was installed, and the sensory blockade reached T_5_.

Seven minutes after administration of anesthesia, the first hypotensive episode appeared, but it was responsive to Ephedrine bolus administration. The hypotension was not associated with other clinical signs or symptoms. After the delivery of the placenta and administering the test dose of Cefazoline (100 mg), sudden circulatory collapse appeared along with deteriorating neurological status and critical respiratory distress with significant desaturation. Urgent supportive therapeutic measures were started: airway management with orotracheal intubation and mechanical ventilation, central venous line placement, vasopressor support with norepinephrine, hemorrhage monitoring, and cardiac evaluation. Blood for cardiac markers was drawn, and an ECG and Echocardiography were performed. The cardiac workup came back normal in all stages. Still, the patient remained severely hypotensive and tachycardic even though the norepinephrine infusion continued with increasing doses (up to 0.6 mcg/kg/min), and the ETCO_2_ was continuously low (between 22–24 mmHg). Hemorrhage was as expected for a surgical delivery.

**Fig. 1.**
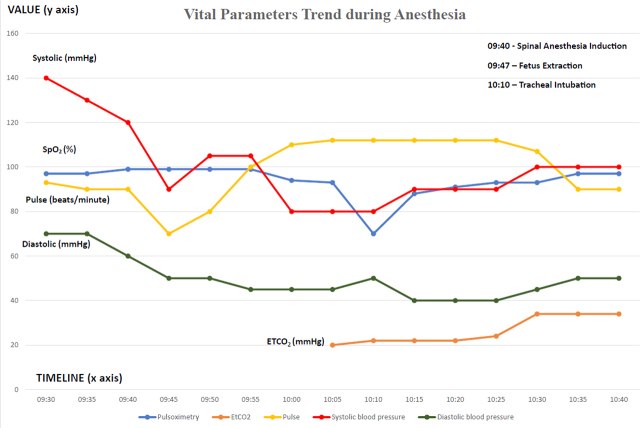
Vital parameters trend during anesthesia

As the cardiovascular status did not significantly change with vasoactive support, two diagnoses came up: amniotic fluid embolism and anaphylactic shock. Even though our patient had several risk factors for AFE (advanced age, hydramnios, cesarean section, male baby), it is a scarce appearance and an exclusion diagnosis. As a consequence, another vasoactive and inotropic positive infusion is started: Epinephrine. Thirty minutes after the collapse’s onset and the vasopressor support initiation, the first bolus dose of Epinephrine was administered (100 mcg), and the arterial blood pressure had a positive response. Cardiovascular stability was achieved only after initiating continuous infusion with Adrenaline (0.04 mcg/kg/min). The surgical delivery was completed 45 minutes after the lumbar puncture, and the patient was transferred to the Intensive Care Unit. At this point, cutaneous eruption appeared at the level of the limbs, Quincke edema, and pruritus. She was also administered hydrocortisone (200 mg). The hemodynamic and respiratory status from the intraoperative period is depicted in Fig. 1 (translation of the anesthesia record)

In the next 4 hours, the patient was weaned off Norepinephrine and the ventilator and was extubated. She was conscious, breathing quietly with good oxygenation. The Adrenaline infusion was administered for the next 17 hours with a low dosage (0.01 mcg/kg/min) and then stopped without hemodynamic consequences.

In this case, the maternal outcome was favorable, the lady being discharged the next day after C-section from the ICU and the third day from the ward to home. She was advised to seek counseling with an immunologist and allergology specialist to investigate antibiotic allergies or other potential substances that can have adverse immunological effects.

## Discussions

Several medical and obstetrical conditions can lead to collapse during labor or delivery.

Our patient presented with a sudden circulatory and respiratory collapse onset during uneventful spinal anesthesia and fetal extraction through transversal cesarean section. The first thing considered was an unwanted high level of neuraxial anesthesia excluded by rechecking the sensory level. The absence of previous cardiac disease and the urgent cardiac examination excluded an acute cardiac event: an ischemic or fatal arrhythmia.

Amniotic fluid embolism (AFE) is a rare but potentially lethal complication that can occur in the peripartum period, a life-threatening anaphylactic reaction caused by amniotic fluid and fetal debris entering the maternal bloodstream [3]. As AFE is a challenging clinical diagnostic, usually one of exclusion, it isn’t easy to estimate the true incidence of the condition. It varies between 1.7 and 7.7 per 100,000 deliveries [4,5,6]. AFE typically occurs during labor or within 30 minutes of delivery of the placenta. Still, in rare cases, it can also occur in any trimester of the pregnancy, during pregnancy termination, or amniocentesis [7].

Even though this condition was first reported more than 100 years ago by Meyer and described in 1941 by Steiner and Lushbaugh, the pathophysiology of AFE still needs to be understood entirely [8,9]. A prerequisite for AFE is the exposure of maternal blood circulation to amniotic fluid or fetal antigens. Two main theories are proposed to explain the mechanism of occurrence: mechanical and immune-mediated [10].

Identified risk factors are classified into maternal (maternal age over 35 years, multiparity, intense contraction during labor, abdominal trauma, cesarean section, induction of labor, placenta praevia, eclampsia, multi-pregnancy, tears in the uterus or cervix, early separation of placenta from the uterus wall) and fetal (fetal distress, fetal death, male baby) [11,12].

Though the clinical picture of our patient is almost superimposed on that of AFE, an essential element is still missing: cataclysmic hemorrhage secondary to coagulation disorders. Moreover, after the patient started developing the rash and the Quincke edema, another cause of the collapse was thought to be the probable cause of the collapse: anaphylactic shock. Even though the event’s severity was extreme, once the appropriate treatment was administered, the cardiovascular status became more balanced, and the patient’s clinical evolution was good.

Many other conditions can lead to cardiorespiratory collapse as the pregnant woman can be subject to several pregnancy-associated diseases and some unrelated to pregnancy. Of these, severe hypertension complicated with eclampsia can be associated with neurological signs and symptoms, but the blood pressure tends to be very high. From the conditions related to low blood pressure or even collapse, hypovolemia due to postpartum hemorrhage [1] is the most frequent complication, which can be controlled with pharmacologic measures (Oxytocin, Ergometrin, Carbetocin, Misoprostol and medication with action on the coagulation) and surgical or obstetrical measures (uterine massage, uterine arteries ligation, B-Lynch sutures, and even hemostatic hysterectomy). Some conditions negatively influence cardiac contractility: peripartum cardiomyopathy, myocardial infarction, pulmonary embolism, and aortic dissection.

As severe anaphylaxis or anaphylactic shock is considered a rare occurrence in pregnancy, it may go easily unsuspected because of multiple pregnancy-related or unrelated conditions that can mimic it [13] and also secondary effects of spinal anesthesia, such as sympathetic antagonism.

The hallmarks of anaphylactic shock are cardiovascular collapse, bronchospasm, cutaneous-mucous signs, and incriminating substances [14]. These signs usually appear in the first minutes after exposure to a specific allergen. The inaugural event is usually the circulatory and respiratory collapse. The skin rash can appear only later after normalizing hemodynamics and respiratory status, so that a quick etiological diagnosis can be difficult. Management of anaphylaxis in pregnancy or immediately after delivery does not differ from the general guidelines [13,14]. The mainstay of treatment in anaphylactic shock is the withdrawal of the incriminated agent and administration of Adrenaline. Any delay in administering adrenaline can lead to worse maternal outcomes and thus should be at all costs avoided [15]. Along with the catecholamine administration, oxygen therapy, and intravenous fluid loading should be carried out simultaneously as they are the most critical adjuncts in treatment. Most of the anaphylactic cases that occur in pregnancy are due to the administration of prophylactic antibiotics [14,16,17].

ACOG recommended the prophylactic administration of the antibiotic within 1 hour of the start of the C-section [18, 19]. Our institutional procedure is administering antibiotics after clamping the umbilical cord to avoid fetal antibiotic exposure. In this case, it was a fortunate timing of administration, as the decreased placental perfusion did not compromise the fetus, as he was already delivered. Whatever the choice for administering the antibiotic, the anesthetist must be fully aware of the potentially fatal complications secondary to this kind of treatment, even though they are rare.

## Conclusion

Even though anaphylaxis and amniotic fluid embolism may result in respiratory distress, hypotension, and cardiovascular compromise, their associated mechanisms, risks, and management strategies are significantly different. Accurate differentiation is crucial for the appropriate treatment of patients and for improving their outcomes. Our case emphasizes the importance of early differencing between anaphylaxis and amniotic fluid embolism to manage the situation as soon as possible. In this specific case, even though a careful history of the mother was taken to identify a potential risk for anaphylaxis, the availability of resuscitation and monitoring equipment and administration of protocol-guided medication improved the maternal outcome, as the lady did not suffer any short-term and long-term sequelae.

## Abbreviations

AFE: (amniotic fluid embolism)
C-section: (cesarean section)
2G2P: (secundi Gesta secundi Para)
mcg: (microgram)
kg: (kilogram)
min: (minute)
iv: (intravenous)
ETCO_2_: (end-tidal carbon dioxide)
mmHg: (millimeters Mercury)
ACOG: (American College of Obstetricians and Gynecologists)
